# Administration of Naloxone by Prehospital Personnel: A Retrospective Analysis

**DOI:** 10.7759/cureus.5602

**Published:** 2019-09-09

**Authors:** Kaitlin M Bowers, Judd Shelton, Eric Cortez, Robert Lowe, John Casey, Andrew Little

**Affiliations:** 1 Emergency Medicine, Hilton Head Hospital, Hilton Head Island, USA; 2 Emergency Medicine, OhioHealth Doctors Hospital, Columbus, USA; 3 Emergency Medicine, OhioHealth Doctors Hospital, Columbus , USA

**Keywords:** opiate abuse, naloxone, prehospital care, ems, overdose

## Abstract

Introduction

Patient-specific discrepancies in prehospital naloxone administration have been documented. As the opioid epidemic continues to evolve, further evaluation of prehospital naloxone administration practices is needed. The objective of this study was to compare patients who received prehospital naloxone and received an emergency department (ED) diagnosis of opioid overdose with patients who received prehospital naloxone and received an alternative ED diagnosis.

Methods

This was a retrospective, multicenter chart review of patients who received naloxone by prehospital personnel for suspected opioid overdose between October 1, 2016, and October 31, 2017. Patients were excluded if age was less than 18 years, naloxone was administered by non-emergency medical service (EMS) personnel, not transported, or if prehospital records could not be linked with ED records. Demographic information and several prehospital clinical findings, including unresponsiveness, apnea, and miosis, were compared between patients diagnosed with opioid overdoses versus an alternative ED diagnosis. Descriptive statistics were utilized.

Results

A total of 837 patients had complete data available and were included in the analysis. Overall, 402 (48%) of patients received an ED diagnosis of opioid overdose, and 435 (52%) of patients received an alternative ED diagnosis. Patients in the alternative diagnosis group were older, had less known drug use, were more likely to be admitted, and had lower incidences of apnea, unresponsiveness, and miosis. In the opioid overdose group, there was a higher proportion of previous drug use, apnea, unresponsiveness, and miosis in the EMS setting, whereas there was a higher proportion of previous overdose, previous suicide attempts, and neurological deficits in the ED setting.

Conclusions

In this retrospective review evaluating patients who received prehospital naloxone, several demographic and clinical differences were noted between the two groups. Further elucidation of the safety and efficacy of prehospital naloxone in alternative diagnoses is needed.

## Introduction

Opioid overdoses is a major public health problem [1]. The mortality rate from overdoses has increased in recent years, and deaths from unintentional overdoses are now the number one cause of accidental death in the United States [1-2]. In 2010, 16,500 of approximately 40,000 total overdose-related deaths were attributed to opioids [3]. Heroin and prescription opioids are both responsible for the observed mortality increases [4].

Emergency medical services (EMS) systems use naloxone to reverse suspected opioid overdoses in the out-of-hospital environment. Naloxone is a competitive antagonist of mu-opioid receptors, reversing the respiratory depression and mental status changes associated with opioid overdoses [5]. Historically, the intravenous administration of naloxone has been the preferred route due to its rapid onset of action. However, several studies in the out-of-hospital setting have demonstrated the effectiveness of alternative routes of administration [6-11].

Out-of-hospital naloxone administration has expanded to basic life support (BLS) providers and law enforcement officials. Several recent studies have described the safe and effective use of naloxone by BLS providers [12-14]. Law enforcement officials trained in naloxone administration have been shown to appropriately recognize opioid overdoses and administer naloxone [15]. 

Potential safety concerns have been noted as the use of naloxone expands. The refusal of transport after prehospital naloxone administration is generally considered safe [16-18]. However, conventional opioid overdose characteristics may be altered with the addition of contaminants and the development of high-potency opioids. Prehospital opioid overdoses requiring the re-dosing of naloxone for the reversal of symptoms are increasing, suggesting a higher potency of opioids [19-20]. The effectiveness of intranasal naloxone has also been questioned for non-heroin opioid overdoses, and the need for longer observation periods after naloxone administration may be indicated for higher potency opioids [21-22].

In the public health sphere, the rates of prehospital naloxone administration may be used to estimate opioid overdose rates. Studies evaluating the effectiveness of this surveillance technique demonstrate mixed results [23-25]. In a recent analysis, naloxone administration had low sensitivity and positive predictive value for opioid overdoses [25].

Patient-specific discrepancies in prehospital naloxone administration have also been documented. In a recent retrospective review of fatal opioid overdose, women patients over 50 years of age and those without clear signs of drug abuse were less likely to receive naloxone [26]. In another case series comparing heroin and prescription opioid overdoses, cases involving heroin were more likely to receive naloxone and less likely to be intubated [27]. Differences in provider perception of treatment and outcomes of overdose-related cardiac arrests compared to non-overdose cardiac arrests have also been noted [28].

As the opioid epidemic continues to evolve, further evaluation of prehospital naloxone administration practices is needed. The objective of this study was to compare patients who received prehospital naloxone and received an emergency department (ED) diagnosis of opioid overdose with patients who received prehospital naloxone and received and an alternative ED diagnosis.

## Materials and methods

This study was a retrospective chart review of patients who received naloxone from prehospital personnel for suspected opioid overdose and were transported to one of three hospital EDs within the same healthcare system. The hospitals involved were in an urban setting with a prevalence of fire-based EMS agencies. The study period was October 1, 2016, through October 31, 2017. All patients 18 years of age and older were included. Patients were excluded if they were younger than 18 years of age, naloxone was administered by non-EMS personnel, or if prehospital records could not be linked with hospital records.

Records from local EMS agencies were reviewed and used to identify patients that received prehospital naloxone and were transported to one of three emergency departments. Using demographic information from EMS records, patients were linked to the healthcare system’s electronic medical record. Patients that could not be linked were excluded from the study.

A datasheet was developed and data points were predefined. Data extractors were trained before reviewing patient care records and supervised by the study authors. Study authors were available for consultation during chart review and held regular meetings to monitor the performance of data extractors. Interrater reliability analysis was not performed.

Information extracted from EMS patient care records included dispatch type, presenting complaints, vital signs, physical examination findings, including pupillary examination and assessment of the level of consciousness, indication for naloxone administration, the total dose of naloxone administered, and subjective response of the patient to naloxone treatment. Data collected from the ED encounter included chief complaint, clinical history, vital signs, physical examination findings, including pupillary examination and assessment of the level of consciousness, the dose of naloxone administered in the emergency department, final ED diagnosis, and final ED disposition. The final ED diagnosis was determined by reviewing the documented diagnosis, laboratory findings, and medical decision-making of the ED provider. Final ED diagnoses were dichotomized into opioid overdoses and alternative diagnoses (all other diagnoses other than opioid overdoses).

Demographic information and several prehospital clinical findings, including unresponsiveness, apnea, or inadequate respiration, and miosis, were compared between the two groups. Apnea was identified as a respiratory rate of zero; inadequate respirations were defined as sonorous respirations, agonal respirations, or the performance of bag valve mask ventilation. Miosis and unresponsiveness were identified by reviewing physical examination findings.

Descriptive statistics were utilized. Data were reported as proportions with 95% confidence intervals, means with standard deviations, and medians with interquartile ranges. Significant testing was performed using chi-square tests, t-tests, and McNemar tests. Statistical significance was defined as p < 0.05.

## Results

A total of 895 prehospital patients were administered naloxone during the study period. A total of 837 patients had complete data available and were included in the analysis. Demographic information and prehospital clinical findings are included in Table 1. Overall, 402 (48%) of patients received an ED diagnosis of opioid overdose, and 435 (52%) of patients received an alternative ED diagnosis.

**Table 1:** Patient demographic information and prehospital clinical findings in the opioid overdose group versus alternative diagnosis group based on final emergency department diagnosis

	Opioid Overdose (N = 402)	Alternative Diagnosis (N = 435)	p-value
Age, Mean ± SD	37.3 ± 12.8	49.7 ± 18.1	<0.001*
Gender, n (%)			
Male	247 (61.4)	253 (58.2)	0.330**
Female	155 (38.6)	182 (41.8)	
Reason for arrival, n (%)			
Unresponsive	114 (28.4)	128 (29.4)	<0.001**
Overdose	109 (27.1)	27 (6.2)	
Cardiac Arrest	92 (22.9)	60 (13.8)	
Other	87 (21.6)	220 (50.6)	
Indication for Naloxone			
Known drug use	284 (71.4)	153 (35.9)	<0.001**
Altered mental status	114 (28.6)	272 (63.8)	
Disposition, n (%)			
Admitted	85 (21.2)	274 (63.1)	<0.001**
Discharged	311 (77.4)	121 (27.8)	
Expired	6 (1.5)	40 (9.2)	
Physical Exam, n (%)			
Apnea	215 (53.5)	121 (27.8)	<0.001**
Unresponsive	294 (73.1)	205 (47.1)	<0.001**
Neurologic deficit	0 (0.00)	17 (3.9)	--
Pinpoint pupils	270 (67.2)	190 (43.7)	<0.001**
* Independent sample t-test ** Chi-square test			

Patients in the alternative ED diagnosis group were older, had less known drug use, were more likely to be admitted, and had lower incidences of apnea, unresponsiveness, and miosis (Table 1). In the opioid overdose group, there was a higher proportion of previous drug use, apnea, unresponsiveness, and miosis in the EMS setting, whereas there was a higher proportion of previous overdose, previous suicide attempts, and neurological deficits in the ED setting (Table 2).

**Table 2:** Comparison of medical history and physical exam findings as gathered by emergency medical services (EMS) and emergency department (ED) personnel for patients with an alternative ED diagnosis

	N = 435	EMS	ED	p-value*
Past medical history, n (%)				
Drug use	426	200 (46.9)	173 (40.6)	0.013
Overdose	407	6 (1.5)	37 (9.1)	<0.001
Suicide Attempt	401	25 (6.2)	39 (9.7)	0.01
Physical Exam, n (%)				
Apnea	435	121 (27.8)	92(21.1)	0.001
Unresponsive	435	205 (47.1)	152 (34.9)	<0.001
Neurologic deficit	386	17 (4.4)	169 (43.8)	<0.001
Pinpoint pupils	405	190 (46.9)	43 (10.6)	<0.001
*McNemar test p-value				

## Discussion

In this retrospective review of patients that received naloxone in the prehospital setting, several demographic and clinical differences were noted between opioid overdose and non-opiate overdose patients. Over half of all patients that received prehospital naloxone were subsequently diagnosed with an alternative diagnosis in the ED. Clinically, alternative diagnosis patients demonstrated lower incidences of apnea, unresponsiveness, and miosis in the prehospital setting as compared to opioid overdose patients.

The decision to administer naloxone in the prehospital settings requires clear indications, a well-defined therapeutic end-point, and a thorough understanding of potential adverse events. In this study, indications for prehospital naloxone administration included suspected opioid overdose and undifferentiated unconscious patients. These indications were included in the EMS agencies’ medical protocols and indirect medical control procedures.

The findings of this study raise several questions about prehospital naloxone indications. In patients exhibiting the classic opioid overdose syndrome, the decision to administer naloxone is relatively straightforward. However, patients presenting with partial toxidromes, mixed toxidromes, or undifferentiated altered mental status may require a more critical risk-benefit analysis. Existing literature is unclear regarding the adverse effects of opioid reversal in the setting of polypharmacy, polysubstance abuse, and concomitant medical and traumatic processes.

The therapeutic endpoint of naloxone therapy is the reversal of apnea and respiratory depression. As its use increases, the risk-benefit ratio of prehospital naloxone administration may need to be re-evaluated in patients without respiratory depression or apnea. Adverse events, such as pulmonary edema and severe agitation following opioid overdose reversal, have been documented [29]. Furthermore, achieving a complete reversal of altered mental status may lead to increased refusals of transport and increased liability for EMS providers and agencies.

The incidence of suspected prehospital opioid overdose may not correlate with the rate of opioid overdoses diagnosed in the ED. In this study, 48% of patients who received naloxone were ultimately diagnosed with an opioid overdose. A proportion of patients who received an alternative ED diagnosis may have received naloxone through unknown unconscious protocols. However, some patients may have received naloxone in the setting of an alternative medical or traumatic process. Although this study did not evaluate the sensitivity or positive predictive value of naloxone administration for opioid overdose, our results are similar to the low sensitivity and positive predictive value reported by Grover et al. [25].

EMS naloxone administration may be used to inform community stakeholders, public safety personnel, and public health officials about opioid overdose locations, patterns, and trends [23-25]. While this process has several advantages, care must be taken to evaluate the final ED diagnoses when extrapolating naloxone administration information to estimate opioid overdose rates as demonstrated by Lindstrom et al. [24].

Several of the patients in this study received multiple doses of naloxone. Recent literature has documented an increase in the requirement for multiple naloxone doses in the prehospital settings [19-20]. While increasing doses and dosing frequencies may be required for more potent and longer-acting opioids, partial responses to naloxone may indicate the presence of other toxins or etiologies of symptoms.

Our study was limited in certain aspects. First, study participants may not be fully representative of the patient population for several reasons. Patients were excluded if there were incomplete data sets, if they were not transported to the ED after prehospital naloxone administration, and if they were transported to another ED not included in the study. Second, this was an urban study setting with a preponderance of fire-based EMS agencies. Therefore, generalizability may be limited in certain circumstances. Third, we amalgamated several data elements to determine the final ED diagnosis. A proportion of this data retrieval was subjective, but we assured consistency in data extraction by direct supervision and review by study authors (KB, JS, AL). Fourth, this was a retrospective study with inherent limitations. However, a datasheet was developed a priori, predefined data points were utilized, and chart reviewers were trained and supervised by study investigators.

## Conclusions

In this retrospective chart review evaluating patients who received prehospital naloxone, several demographic and clinical differences were noted between patients diagnosed with opioid overdoses versus an alternative ED diagnosis. Further elucidation of the safety and efficacy of prehospital naloxone in alternative ED diagnosis patients is needed.
